# Associations between risk factors in childhood (12–13 years) and adulthood (48–49 years) and subclinical atherosclerosis: the Kaunas Cardiovascular Risk Cohort Study

**DOI:** 10.1186/s12872-015-0087-0

**Published:** 2015-08-18

**Authors:** Indre Ceponiene, Jurate Klumbiene, Egle Tamuleviciute-Prasciene, Justina Motiejunaite, Edita Sakyte, Jonas Ceponis, Rimvydas Slapikas, Janina Petkeviciene

**Affiliations:** Department of Cardiology, Medical Academy, Lithuanian University of Health Sciences, Eiveniu 2, Kaunas, LT-50009 Lithuania; Faculty of Public Health, Medical Academy, Lithuanian University of Health Sciences, Siaures av. 57, Kaunas, LT-49264 Lithuania; Department of Endocrinology, Medical Academy, Lithuanian University of Health Sciences, Eiveniu 2, Kaunas, LT-50009 Lithuania

## Abstract

**Background:**

The data on the childhood determinants of adult cardiovascular disease (CVD) are lacking in populations of Eastern Europe that are characterised by substantially high CVD mortality. From a public health perspective, it is important to identify high-risk individuals as early as possible in order to have the greatest benefit of preventive interventions. The aim of this study was to evaluate the associations of childhood and adulthood traditional risk factors with subclinical atherosclerosis and arterial stiffness in a Lithuanian cohort followed up for 35 years.

**Methods:**

The study cohort consisted of 380 adults aged 48–49 from Kaunas Cardiovascular Risk Cohort study, who were followed up since childhood (12–13 years). The baseline survey (1977) included blood pressure (BP) and anthropometric measurements and sexual maturity scale. In the follow-up survey (2012), BP, anthropometric and lipids measurements, interview about smoking, measurement of carotid intima-media thickness (IMT) and determination of pulse wave velocity (PWV) were performed. Two types of general linear models were applied to test the associations of childhood and adulthood risk factors with IMT and PWV. Model 1 included only childhood variables. In model 2, adulthood variables were added to childhood variables.

**Results:**

In linear regression model with childhood variables childhood systolic BP (β = 0.014; p = 0.016) and BMI (β = 0.006; p = 0.003) were directly associated with IMT only in women. When adulthood variables were included into regression model, the association between childhood systolic BP and IMT remained significant (β = 0.013; p = 0.021), while childhood BMI was not associated with IMT (β = 0.003; p = 0.143). Additionally, association of adult smoking and IMT was found in women (β = 0.033; p = 0.018). IMT of men was directly related to adult systolic BP (β = 0.022; p = 0.018) and inversely to HDL cholesterol level (β = −0.044; p = 0.021). PWV was directly associated only with adult systolic BP in both genders (β = 0.729 for men and β = 0.476 for women; p = 0.001).

**Conclusions:**

Sex differences in the associations between childhood and adulthood risk factors and subclinical atherosclerosis were found. The results of the study support efforts to reduce conventional risk factors both in childhood and adulthood for the primary prevention of atherosclerosis.

## Background

Atherosclerotic diseases such as coronary heart disease, stroke and peripheral artery disease are great threats to public health in Lithuania which has one of the highest cardiovascular mortality in Europe [1]. The process of atherosclerosis begins early in childhood and typically remains asymptomatic until later in life. The cardiovascular risk factors induce changes in the arterial wall that lead to stiffening and atherosclerotic plaque formation. Carotid intima-media thickness (IMT) demonstrates structural changes in the arterial wall even in early subclinical stages of atherosclerosis. IMT is directly related to increased cardiovascular risk and can predict cardiovascular events, such as myocardial infarction or stroke in asymptomatic adults [2]. Prospective cohort studies have demonstrated that blood pressure, lipid levels, body mass index (BMI) and some health behaviours, identified in childhood, predict increased IMT in adulthood [3–6]. Childhood risk factors measured at or after the age of 9 showed the strongest associations with subclinical atherosclerosis in adulthood [7].

Arterial stiffness expressed as pulse wave velocity (PWV) is a strong predictor of future cardiovascular events [8–10]. PWV is associated with subclinical target organ damage in the coronary, peripheral arterial, cerebral, and renal arterial beds [11]. Previous observations concerning the relationship between risk factors in childhood and arterial stiffness have been controversial. In the Bogalusa Heart Study and in the Cardiovascular Risk in Young Finns Study, systolic BP in childhood was directly associated with PWV in adulthood, whereas in the Atherosclerosis Risk in Young Adults (ARYA) Study no association between childhood BP and adult PWV was found [12–14]. Controversy in the results from these studies might be due to differences in methodology of PWV measurement and different age of the participants during the last follow-up (27–30 years in the ARYA study, compared to 30–45 years in the Young Fins study and 24 to 44 years in the Bogalusa study).

Population-based data from prospective cohort studies linking childhood risk factors with adulthood risk factors and preclinical markers of cardiovascular health are of particular importance because they help to identify individuals at high CVD risk. Such data are lacking in populations of Eastern Europe that are characterised by considerably high CVD risk profile and mortality rates and one of the highest male–female differences in cardiovascular health in Europe [15]. From a public health perspective, high priority should be given for early prevention of CVD in those countries including Lithuania. Preventive interventions are more likely to be successful when aimed at individuals who have an increased risk of developing CVD.

The aim of this study was to determine the associations of childhood and adulthood traditional risk factors with subclinical atherosclerosis and arterial stiffness in a Lithuanian cohort followed up for 35 years.

## Methods

### Study design and sample

This study used data from the Kaunas Cardiovascular Risk Cohort study, a prospective study with the baseline data collected in 1977 on a random sample of 1082 Kaunas schoolchildren aged 12–13 years [16, 17]. Over the period of follow-up, 8.4 % (n = 91) individuals died, 9.5 % (n = 103) emigrated from Lithuania, 0.4 % (n = 4) were severely ill and the addresses of 8.3 % (n = 90) subjects were not available in the National Population Register. In 2012, 507 participants (63.9 % of eligible sample) aged 48–49 were surveyed; however, only individuals who had both vascular ultrasound and pulse wave measurements performed were included in the analysis (n = 380).

The study protocol was approved by the Lithuanian Bioethics Committee (permission No. BE-2-30). Written consent on behalf of the children enrolled in the first survey (1977) was obtained from parents or guardians. Written informed consent for the participation in the follow-up survey (2012) was obtained from all participants.

### Measurements in childhood

BP measurements were performed with a standard mercury sphygmomanometer in the sitting position after 5 minutes of rest. BP was measured to the nearest 2 mmHg on the right arm. The first Korotkoff phase was used to determine systolic BP, and the fifth phase was used to determine diastolic BP. Three consecutive BP measurements were taken. The average of these three measurements was used in the analysis.

The height of participants, without shoes, was measured to the nearest centimetre with a stadiometer. The body weight of participants, wearing light indoor clothing and no shoes, was measured to the nearest 0.1 kg with standardised medical scales. BMI was calculated as weight divided by height squared (kg/m^2^).

A modified Tanner scale (excluding examination of the testes) was used for evaluation of sexual maturity. The development of axillary hair and pubic hair for girls and boys, growth of moustache for boys, and the development of breast and menstruation for girls were assessed for calculation of sexual maturity score [16]. The sexual maturity scores could vary from 0 to 12 in boys and from 0 to 15 in girls (the higher numbers mean higher sexual maturity).

### Measurements in adulthood

BP, height and weight were measured using the same methodology as in childhood. Blood samples for lipids measurements in adulthood were taken in the morning after fasting at least 12 hours. All measurements were performed in a certified laboratory on an automatic analyser Cobas Integra 400 plus. Serum lipid levels were determined using conventional enzymatic methods.

Information on cigarette smoking was collected by a standard questionnaire. Participants were divided in daily smokers and others. Level of education was determined by the following question: ‘What is your education?’ Possible answer choices were: 1) incomplete secondary, 2) secondary, 3) vocational school, 4) college, 5) university. Participants were categorized into three educational groups: low education (incomplete secondary or secondary education), intermediate education (vocational school), and high education (college or university).

### Carotid intima-media thickness measurement

Carotid ultrasonography to assess IMT of the common carotid artery was performed using high-resolution B-mode ultrasound 7 MHz vascular probe (Vivid 7®, GE). During the exam patient was in supine position; the patient’s neck was positioned in hyperextension and slightly inclined at 45°. IMT measurement was carried out in the anterior and posterior wall of both common carotid arteries, at a distance of 1–1.5 cm from the carotid bifurcation. A 10-mm-long segment of the region of interest was placed manually for detection of the carotid intima–media [18]. Using a software package for semi-automated border detection loaded on the system (EchoPAC®, GE), mean carotid IMT values were obtained. Three measurements were taken on each carotid artery, and mean values of IMT were calculated for further analysis.

### Measurement of pulse wave velocity

PWV measurements were performed using the technique of SphygmoCor System (AtCor Medical Pty Ltd., Head Office, West Ryde, Australia) [18, 19]. To ensure haemodynamic stability, the measurements were performed in the supine position after the participants had been resting in this position for a minimum of 10 minutes. All subjects were familiarised with the environment, the procedure, and the devices. After 10 min of rest in the supine position, the brachial artery BP was recorded twice consecutively with a 1 min interval between each measurement with a mercury sphygmomanometer using the auscultatory method. The PWA profile was obtained by placing tonometer over the radial artery.

PWV was measured estimating the delay in pulse wave at carotid and femoral level as compared to the electrocardiogram wave. The distance from the carotid site to the suprasternal notch (proximal distance) and the distance from the suprasternal notch to the femoral site (distal distance) were measured. The path length was calculated as the difference between the distal and proximal distances. The tonometer probe was placed at the right carotid and femoral arterial sites subsequently. PWV was calculated automatically as the carotid-femoral path length in meters divided by the carotid–femoral transit time in seconds using the intersecting tangent algorithm. To account for a distance measurement error, ‘real’ PWV was calculated by multiplying obtained PWV by 0.8 [20, 21].

Fifty measurements of IMT and PWV were assessed by a second investigator with between-observer coefficient of variation 4 % for IMT and 6 % for PWV.

### Statistical analysis

All statistical analyses were performed using statistical software package IBM SPSS Statistics 20. Categorical variables were expressed as percentages and tested by the χ^2^ test. The normality of distribution of continuous variables was tested by Kolmogorov-Smirnov test. Means and standard deviations (SD) were presented for the normally distributed continuous variables while median and interquartile range was calculated for the distributions that did not meet the criteria of normality. Student t test was used to compare the mean values of normally distributed variables and Mann–Whitney test was applied for the comparison of non-normal distributions.

General linear models (GLM) were applied to test the effect of childhood and adulthood risk factors on IMT and PWV. Two types of models were fitted. Model 1 included childhood systolic BP, BMI and sexual maturity score. Effect modification by sexual maturity score was examined by adding interaction terms of the score with systolic BP and BMI to the model. In model 2, adulthood variables (systolic BP, BMI, high-density lipoprotein (HDL) cholesterol, low-density lipoprotein (LDL) cholesterol, smoking, and educational level) were added to childhood variables.

All analyses were performed separately for men and women. P values of less than 0.05 were considered statistically significant.

## Results

The characteristics of the study participants are presented in Table 1. In childhood, systolic BP and BMI were significantly higher in girls than in boys. The degree of sexual maturation differed between girls and boys. Girls were more sexually mature than boys. The proportion of girls who had reached menarche was 37.7 %. They had higher systolic BP (121.8 (13.8) mmHg) and BMI (21.1 (3.4 kg/m^2^) then those who were less sexually mature (114.3 (11.0) mmHg and 18.3 (2.7) kg/m^2^ respectively; p < 0.001).

**Table 1:** Characteristics of the study population in childhood and adulthood

Characteristic	Men	Women	P value
	n = 168	n = 212	
Childhood (12–13 years)			
Systolic BP, mm Hg (mean; SD)	112.3 (10.7)	116.6 (12.4)	0.001
Diastolic BP, mm Hg (mean; SD)	55.1 (10.2)	55.6 (11.1)	0.649
BMI, kg/m^2^ (median; IQR)	17.9 (16.5;20.0)	18.6 (17.1;20.2)	0.021
Sexual maturity score (median; IQR)	0 (0;1)	6 (4;9)	<0.001
Adulthood (48–49 years)			
Systolic BP, mm Hg (median; IQR)	134.3 (123.5;150.0)	125.6 (116.2;138.7)	<0.001
Diastolic BP, mm Hg (median; IQR)	89.3 (82.0;96.0)	80.7 (74.8;88.0)	<0.001
LDL cholesterol, mmol/l (mean; SD)	4.0 (1.1)	3.8 (1.1)	0.106
HDL cholesterol, mmol/l (mean; SD)	1.4 (0.5)	1.8 (0.4)	<0.001
BMI, kg/m^2^ (median; IQR)	26.7 (24.5;29.5)	25.7 (23.0;29.6)	0.145
Hypertension, N (%)	106 (63.1)	77 (36.3)	<0.001
Smoking, % N (%)	68 (40.5)	41 (19.3)	<0.001
Intima- media thickness, mm (mean; SD)	0.66 (0.11)	0.61 (0.08)	<0.001
Pulse wave velocity, m/s (mean; SD)	7.1 (2.1)	6.1 (1.7)	<0.001
Educational level, N (%)			
Low	54 (32.1)	47 (22.2)	0.058
Intermediate	53 (31.5)	67 (31.6)	
High	61 (36.3)	98 (46.2)	

Abbreviations: *BMI* body mass index; *BP* blood pressure; *HDL* high density lipoprotein; *IQR* interquartile range (25 percentile and 75 percentile values); *LDL* low density lipoprotein; *SD* standard deviation

Adult men had worse cardiovascular risk profile compared to women. Systolic and diastolic BP, also the prevalence of hypertension and smoking were higher, whereas levels of HDL cholesterol were lower in men than in women. Men had higher values of IMT and PWV.

The linear regression model with childhood variables (model 1) showed that childhood systolic BP and BMI were directly associated with IMT only in women (Table 2). No interaction between female sexual maturity score and systolic BP (p-value of interaction term 0.231) or BMI (p-value of interaction term 0.833) on IMT was found. When adulthood variables were included into model 2, the association of childhood BMI with IMT of women was attenuated, while the contribution of childhood systolic BP remained significant (β = 0.013; p = 0.021). Interestingly, association of adult smoking and IMT was significant in women (β = 0.033; p = 0.018), but not in men. IMT of men was directly related to adult systolic BP (β = 0.022; p = 0.018) and inversely to HDL cholesterol level (β = −0.044; p = 0.021). No associations between adult BMI as well as LDL cholesterol level and IMT were found in both genders. Analysed childhood and adulthood variables explained 10 % of variation of IMT in men and 13 % in women.

**Table 2:** Associations between intima-media thickness and variables measured in childhood and adulthood

Variable	Men			Women		
	β	95 % CI	P value	β	95 % CI	P value
Model 1						
Childhood systolic BP, mm Hg (for a 1-SD change)	0.001	−0.001;0.003	0.357	0.014	0.003;0.025	0.016
Childhood BMI kg/m^2^	−0.002	−0.008;0.004	0.514	0.006	0.002;0.010	0.003
Sexual maturity score	−0.004	−0.016;0.009	0.570	−0.003	−0.006;0.001	0.183
R^2^	0.008			0.082		
Model 2						
Childhood systolic BP, mm Hg (for a 1-SD change)	0.002	−0.020;0.023	0.867	0.013	0.002;0.025	0.021
Childhood BMI kg/m^2^	−0.003	−0.10;0.004	0.380	0.003	−0.001;0.008	0.143
Sexual maturity score	−0.001	−0.014;0.011	0.853	−0.003	−0.007;0.001	0.147
Adult systolic BP, mm Hg (for a 1-SD change)	0.022	0.004;0.041	0.018	0.006	−0.006;0.018	0.320
HDL cholesterol mmol/l	−0.044	−0.082;-0.007	0.021	0.011	−0.017;0.040	0.431
LDL cholesterol mmol/l	0.002	−0.014;0.017	0.844	0.004	−0.006;0.014	0.390
Adult BMI, kg/m^2^	0.002	−0.002;0.006	0.363	0.002	−0.001;0.005	0.114
Daily smoking	0.006	−0.003;0.041	0.755	0.033	0.006;0.06	0.018
Education	0.001	−0.022;0.021	0.991	0.007	−0.006;0.021	0.292
R^2^	0.104			0.133		

Reference group for daily smokers was nonsmokers and occasional smokers.

Abbreviations: *BMI* body mass index; *BP* blood pressure, *CI* confidence interval; *HDL* high-density lipoprotein; *LDL* low-density lipoprotein

Linear regression analyses (model 1) did not demonstrate any association of childhood BP and childhood BMI with PWV in men and women (Table 3). Adult systolic BP was significantly associated with PWV in both genders (β = 0.729 for men and β = 0.476 for women; p = 0.001) (model 2). PWV was not related to adult BMI, lipid levels, and smoking. Variables included into model 2 explained 12 % of variation of PWV in men and 13 % in women.

**Table 3:** Associations between pulse wave velocity and variables measured in childhood and adulthood

Variable	Men			Women		
	β	95 % CI	P value	β	95 % CI	P value
Model 1						
Childhood systolic BP, mm Hg (for a 1-SD change)	0.221	−0.190;0.631	0.290	−0.076	−0.318;0.166	0.537
Childhood BMI kg/m^2^	0.054	−0.070;0.177	0.391	0.028	−0.064;0.121	0.544
Sexual maturity score	0.079	−0.184;0.342	0.553	0.102	0.015;0.188	0.022
R^2^	0.024			0.046		
Model 2						
Childhood systolic BP, mm Hg (for a 1-SD change)	0.045	−0.372;0.462	0.832	−0.165	−0.409;0.079	0.184
Childhood BMI kg/m^2^	0.069	−0.067;0.206	0.319	−0.003	−0.111;0.105	0.953
Sexual maturity score	−0.001	−0.014;0.011	0.853	0.100	0.014;0.185	0.023
Adult systolic BP, mm Hg (for a 1-SD change)	0.726	0.328;1.123	<0.001	0.476	0.208;0.744	0.001
HDL cholesterol mmol/l	−0.215	−1.028;0.598	0.602	−0.102	−0.727;0.523	0.748
LDL cholesterol mmol/l	−0.049	−0.363;0.266	0.761	−0.033	−0.249;0.183	0.763
Adult BMI, kg/m^2^	0.005	−0.083;0.094	0.903	0.025	−0.036;0.086	0.412
Daily smoking	0.263	−0.440;0.966	0.460	−0.314	−0.918;0.291	0.307
Education	−0.016	−0.434;0.401	0.939	−0.012	−0.318;0.294	0.939
R^2^	0.117			0.133		

Reference group for daily smokers was nonsmokers and occasional smokers.

Abbreviations: *BMI* body mass index; *BP* blood pressure, *CI* confidence interval; *HDL* high density lipoprotein; *LDL* low-density lipoprotein

## Discussion

Our study demonstrated a significant association of childhood systolic BP with adult carotid IMT in women. We did not find any relationship between childhood risk factors and IMT in men. High adult systolic BP and low HDL cholesterol were associated with increased IMT in men. PWV was not related to childhood risk factors. The association between adult systolic BP and PWV was significant in both genders.

Previous cohort studies found that childhood BP was predictive of adulthood vascular changes as measured by carotid IMT [3–5]. The Cardiovascular Risk in Young Finns Study has shown that childhood BP is significantly related to thickness of carotid IMT in adulthood taking into account adult BP levels [3]. However, the pooled analyses of four prospective studies demonstrated that only persistently elevated BP from childhood to adulthood was associated with increased risk of subclinical atherosclerosis, while the individuals with high childhood BP and normal adult BP did not have significantly increased IMT [22]. Our study found that, after adjustment for adult BP, childhood systolic BP was positively associated with carotid IMT in women. This is in line with the observations from Bogalusa Heart Study in which childhood systolic BP was significant predictor of carotid IMT only in white women [23]. The causes of sex differences in the associations between childhood BP and carotid IMT are not studied extensively. Physiological mechanisms underlying the observed sex differences might be related with the influence of sex hormones and other female-specific factors [24]. In baseline survey of our cohort, girls were more sexually mature and had higher values of systolic BP than boys. Meanwhile, the increase in BP from childhood to adulthood was much more pronounced in men than in women. Possibly, in men tracking of BP from childhood to adulthood had greater influence on risk of subclinical atherosclerosis than childhood BP.

Several cohort studies demonstrated that childhood BMI is associated with carotid IMT in adulthood [25–27]. The British cohort study found associations of childhood BMI with IMT in men but not in women, however, the follow-up period was much longer than in our study (till 60–64 years) when men have increased atherosclerotic risk compared to women [27]. The findings of the Bogalusa Heart Study showed that childhood BMI was positively related to adult levels of carotid IMT; however, the association was reduced, after controlling for adult BMI [26]. High IMT levels were observed only among overweight children who became obese adults [28]. Finnish investigators also reported that the association between adolescent BMI and adult IMT became non-significant after adjustment for BMI measured in adulthood [29]. In systematic review on childhood obesity and adult cardiovascular disease risk, Lloyd LJ et al. found little evidence that childhood obesity is an independent risk factor for carotid IMT [30]. The authors argued that the associations might reflect the tracking of BMI from childhood to adulthood. The findings of our study revealed the association of childhood BMI with carotid IMT only in women when the data were not adjusted for adult BMI. The differences in the association of BMI with IMT between genders might be driven by the fact that girls were more mature compared to boys. However, after adjustment, the association became non-significant which is in line with earlier reported data. We did not perform tracking of BMI categories from childhood to adulthood due to small number of study participants in some of the subgroups.

In our study, adult smoking increased IMT in women, but not in men. The recent review of sex differences in cardiovascular risk factors concluded that smoking is significantly more hazardous for CVD developing in women than in men [24]. The mechanisms of such differences are not sufficiently investigated.

Limited data are available regarding the effects of childhood risk factors on adult arterial stiffness. The prospective Cardiovascular Risk in Young Finns Study demonstrated that conventional risk factors in childhood predict PWV in adulthood [13]. Moreover, this study showed that the favourable change in ideal cardiovascular index was inversely related to PWV [31]. This association remained significant after adjustment for the baseline index suggesting that positive changes in lifestyle and risk factors could have favourable impact on the stiffening process of arteries. In our study, childhood BP and BMI were not predictive for PWV, whereas higher adult systolic BP was associated with higher PWV in both genders. Similarly, the recent data from Bogalusa Heart Study showed no association between childhood BP and adult PWV measured 27 years later after adjustment for adulthood BP, although previously this study reported the adverse influence of elevated childhood BP on arterial stiffening process [12, 32].

Summarising the results of most prospective studies, positive associations between childhood risk factors and adult subclinical atherosclerosis were generally attenuated after adjustment for adult CVD risk factors. Those findings do not mean that early CVD prevention and intervention measures during childhood might be irrelevant. Our previous studies confirmed that childhood BP was related with adult hypertension, also childhood BMI was associated with risk of adult obesity, metabolic syndrome, hyperglycaemia and diabetes [17, 33]. A substantial part of individuals with elevated childhood BP and increased childhood BMI had high CVD risk profile in adulthood. Those data emphasize that CVD risk factors should be prevented as early as possible with continuation of health promotion activities throughout the life course. Promotion of healthy nutrition and physical activity in childhood might help to avoid excessive weight gain and to prevent adult high BP. CVD prevention programmes should be targeted to children with higher levels of CVD risk factors to maximize benefit of preventive measures.

The strength of the present study is the use of population-based data from a randomly selected cohort prospectively followed up for 35 years. BP and anthropometric measurements were performed using the same methodology in the first and in the last survey. A further strength is the use of measurements that allow determining early subclinical stages of atherosclerosis strongly related with future cardiovascular events. As a limitation of our study, it should be noted that the relatively small sample size might contribute to the observed sex variations in the associations between childhood and adulthood risk factors and subclinical atherosclerosis. Moreover, the number of risk factors measured in childhood was quite limited. Finally, the loss of participants over 35 years of follow-up was quite substantial. One of the reasons for this was a high rate of emigration from Lithuania over the last decades. Although no differences between participants and non-participants were found in existing baseline measurements [17], the bias due to differential loss to follow-up was possible.

## Conclusions

Sex differences in the associations between childhood and adulthood risk factors and subclinical atherosclerosis were found. In women, childhood BP was predictive of IMT later in life irrespectively of adult risk factors. Vascular health of men was related mainly to adult BP. These results support efforts to reduce conventional risk factors both in childhood and adulthood for the primary prevention of atherosclerosis.

## Abbreviations

CVD: Cardiovascular disease
BMI: Body mass index
BP: Blood pressure, CI, Confidence interval
HDL: High density lipoprotein
IMT: Intima-media thickness
LDL: Low-density lipoprotein
PWV: Pulse wave velocity
